# Flight Altitude of Common Cranes (*Grus grus*) Crossing the Arkona Basin (Baltic Sea): Implications for Offshore Wind Farm Development

**DOI:** 10.1002/ece3.72714

**Published:** 2025-12-12

**Authors:** Henrik Skov, Stefan Heinänen, Lars O. Mortensen, Johan Månsson, Lovisa Nilsson, Rune S. Tjørnløv, Ramunas Zydelis

**Affiliations:** ^1^ DHI Hørsholm Denmark; ^2^ NOVIA, University of Applied Sciences Turku Finland; ^3^ Department of Ecology Swedish University of Agricultural Sciences Riddarhyttan Sweden; ^4^ WSP Taastrup Denmark; ^5^ Ornitela, UAB Vilnius Lithuania

**Keywords:** collision risk, laser rangefinders, long‐distance migration, satellite tracking, weather influence on flight profile

## Abstract

With the planned large‐scale development of offshore wind farms, there is a need for an improved understanding of the potential future interactions between migrating common cranes ( *Grus grus* ) and the wind turbines as they cross areas of open sea during migration. The Arkona Basin is currently the focus of large‐scale offshore wind farm development activities, with full development of the region's capacity for offshore wind projected to cover approximately 80% of the migration corridor. By using laser rangefinder tracking and GPS‐tagged crane individuals, we studied the vertical flight behaviour in relation to weather conditions as they cross the Arkona Basin in the Baltic Sea between Sweden and Germany. The effect of weather conditions on the vertical distribution (i.e., flight altitudes) of the cranes was modelled using generalised additive mixed models. The results show that the flight altitude of common cranes crossing the basin strongly depends on the wind and clearness conditions. Both during the spring and autumn migration, the cranes utilise thermal winds at the coast to soar and frequently reach altitudes > 300 m. Yet, the model predictions showed that the flight altitude descended towards the central offshore parts of the basin targeted for offshore wind farm development, with a steeper descending trend and flight altitudes at rotor height during crosswind and headwind conditions and during poor and moderate clearness (< 60%). Our results indicate that, in combination with their low level of macro avoidance, the overall collision risk of migrating cranes will depend on the frequency of adverse conditions, which cause the birds to fly at rotor height over the wind development zone. Implementation of efficient mitigation measures (e.g., turbine curtailment) to minimise the risk of collision with the future large‐scale wind turbine installations in the region is obviously a conservation priority.

## Graphical abstract

Study comprises the first field study of the flight altitude of the common crane ( *Grus grus* ) during seasonal migrations across the Baltic Sea. Our investigations clearly indicate that despite weather‐induced variations in the flight altitude, the majority of cranes will cross the Baltic Sea at altitudes between 50 and 250 m. As a consequence, future interactions between migrating cranes and offshore wind farm projects should be anticipated, and especially during adverse weather conditions, the cranes are predicted to fly at rotor height over the wind development zone in the region.

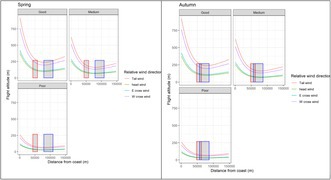

## 1 Introduction

Development of wind energy is among many renewable options to reduce greenhouse gas emissions (Li et al. 2022). However, the expansion of wind energy may have detrimental effects on biodiversity, and one of the main impacts is lethal collisions of birds (Thaxter et al. 2017). Flight altitude constitutes an important element of bird collision risk with wind energy installations (Schaub et al. 2024). Due to the lower potential for soaring over open sea (Newton 2008), soaring species like the common crane (*Grus grus*, hereafter crane) may be exposed to a higher risk of collision with wind energy development offshore. Moreover, bird migration studies by radar and visual observations generally show that wind conditions have a major influence on the altitude of migration, with the height being greater in tail wind conditions than in headwinds (Bruderer 1971, 1997; Kumari 1983; Bruderer and Liechti 1998). Yet, detailed studies of the migratory behaviour of cranes are currently missing from many areas targeted for offshore wind development.

The crane is a short to long‐term migrant using thermal winds and soaring during migration (Alerstam and Bauer 1973; Alerstam 1975). The migration strategy of the cranes is manifested as a strong avoidance of crossing large expanses of open water. This strategy leads to a funnelling effect along well‐established migratory corridors like the Arkona Basin in the western Baltic Sea, where the cross‐sea distance between Sweden and Germany is the shortest. Migration corridors for soaring species of birds are generally assessed as being among the most sensitive to wind energy developments (Gauld et al. 2022). Hence, the Arkona Basin constitutes an important and sensitive migratory corridor for the cranes migrating between wintering grounds in Southwest Europe and breeding grounds in Sweden and Norway (Swanberg 1987). The crane is long‐lived with a high annual survival rate (Bautista and Alonso 2021). Crane populations in northern Europe have shown an increasing trend at least over the past 27 years; an average increase of 0.84% per year from 1988 to 2012 and 2.43% per year from 2003 to 2012 (Wetlands International 2016). The crane population in Sweden is estimated to have increased by 4% annually since 1997 but has stabilised after 2005 (Wetlands International 2016).

When crossing the Arkona Basin, the cranes use the thermal uplifts occurring near the coast to gain altitude to maximum heights around 1000 m before crossing the basin (Mortensen et al. 2020). This is in line with tracking studies from other regions which show frequent soaring heights of the species above 500 m (Pekarsky et al. 2024). However, due to the lack of data from the offshore parts of the region, little is documented on the ability of cranes to capitalise on favourable wind conditions and maintain a high migration altitude as they cross the 100–130 km wide open sea of the Arkona Basin. The importance of establishing the dynamics of the flight altitude of migrating cranes over the Arkona Basin has been emphasised by the observed lack of avoidance of the species towards large‐scale offshore wind farms (Skov et al. 2015; Kulik et al. 2020), and the plans for large‐scale offshore wind farm development in the region (Supporting Information S1). The future wind turbines are expected to be 15 MW+ with a minimum height of 260 m. The 27 projected wind farms will cover 80% of the migration corridor used by cranes (Wind Europe 2025). Despite this, no detailed studies have examined how such development may affect crane migration under varying weather conditions. The main aim of this study was to test the hypothesis that migrating cranes during adverse weather conditions may fly at altitudes corresponding to the rotor height of the future wind turbines when crossing the offshore parts of the Arkona Basin. Accordingly, our study focused on investigating how the flight altitude varies with weather conditions (wind direction and clearness) and distance to shore in the Arkona Basin. We applied GPS satellite tracking to obtain full cross‐sea height profiles from tracked cranes as well as coastal tracking observations using laser rangefinders. The possibility of collecting a large sample size at the coasts as well as offshore enabled us to assess the dynamics of flight altitudes across a wide range of wind and clearness conditions and validate the assumptions concerning elevated collision risk to wind turbines offshore. Hence, the results were anticipated to improve assessments of the potential interactions between cranes and the future offshore wind energy installations in the Baltic Sea.

## 2 Material and Methods

### 2.1 Study Area

**FIGURE 1:**
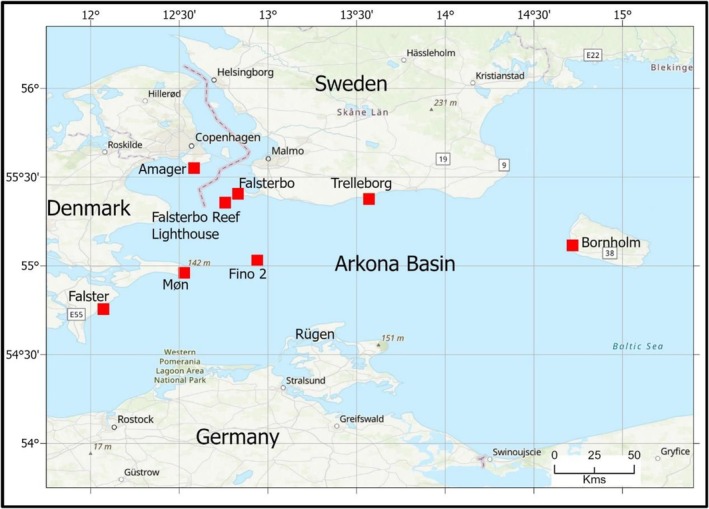
Overview of study area in the Arkona Basin. Location of data collection sites is indicated by red squares.

The Arkona Basin is located in the western Baltic Sea, between the German, Danish and Swedish coasts, north of the island Rügen (Figure 1). The region typically experiences mild winters with temperatures around 0°C and cool summers. The prevailing winds during spring and autumn are from the west and southwest in the area. As the vast majority of the Swedish and Norwegian population of cranes crosses the Arkona Basin during migration (Swanberg 1987; Hansson et al. 2024), the region provides an optimal setting for detailed investigation of cranes' flight altitude profiles. During autumn, cranes leave the southern part of mainland Sweden and cross the Arkona Basin aiming for Rügen, where they usually stop over before continuing south. The Swedish and Norwegian population that passes the Arkona Basin is estimated at 84,000 individuals (Wetlands International 2016), and they mainly cross the region between Bornholm and Falster over a broad front both during spring and autumn (Hansson et al. 2024).

Following energy fuelling periods at autumn and spring staging sites, cranes generally depart from Sweden between mid‐September and mid‐October, and from Rügen, Germany, between mid‐March and mid‐April, followed by the crossing of the Arkona Basin.

### 2.2 Flight Altitude

Flight altitude data of migrating cranes was collected by visual tracking using laser range finders and satellite tracking data derived from GPS‐tagged individuals.

#### 2.2.1 Laser Rangefinder

Laser rangefinder tracking of migrating common cranes was carried out to collect 3‐D flight data from the FINO‐2 research platform in the German part of Krieger's Flak located 40 km from the coasts of Germany and Sweden as well as from the coasts of Denmark and Sweden (Skov et al. 2015; Figure 1; Table 1). All rangefinder tracking locations were located less than 20 m above sea level.

**TABLE 1:** Overview of rangefinder tracking of common cranes.

Period	Location	Latitude/longitude	Sample size (number of tracks)
3 September 3 to 16 October 2013	FINO‐2 platform	55.01/13.15	6
31 March to 21 April 2013 and 11–12 October 2013	Falsterbo Reef Lighthouse	55.19/12.40	14
1 April to 24 May 2013 and 2 October to 11 October 2013	Falster^a^	54.92/12.56	120
23 September 2014	Bornholm	55.12/14.74	8
31 March to 12 May 2013 and 20 September to 24 September 2013	Falsterbo^b^	55.31/13.31	181

^a^ Includes Amager and Møn.

^b^ Includes Trelleborg.

Laser rangefinders of the type Vectronix 21 Aero were used to collect species‐specific 3‐D data on migrating cranes. The laser rangefinder is equipped with a built‐in, battery‐driven laser system that allows recordings of distance, altitude and direction to a given object. Thus, operated at known geographical positions and elevations, the laser rangefinders can be used to obtain 3‐D data on migrating birds. Under optimal conditions, laser rangefinders can be used up to approximately 3 km for the largest bird species, depending on the angle of view and bird flight behaviour (i.e., gliding, soaring, or flapping). Detection bias is inevitable during situations with low visibility (< 1 km), and thus recordings were not undertaken during such conditions.

Laser rangefinders can be operated with repeated recordings at intervals of approximately 10–15 s, with GPS‐logged positions and altitudes automatically recorded. This provides long data series of recordings for individual birds or flocks. In general, as cranes are primarily migrating in flocks rather than individually, the rangefinder recordings covered tracks of flocks. Crane flocks were tracked for periods as long as possible, and with a mean tracking time of 5 min and 18 s. Crane migration mainly took place between 9 am and 5 pm (UTC), and rangefinder tracking was therefore focused on this time period. Similar to detections by binoculars, there is a risk that less accurate rangefinder readings will take place if gliding birds are moving directly towards or away from the observer. The metal constructions on the FINO‐2 platform and the Falsterbo light house caused distortion of the collected GPS data by the rangefinder, which may have interfered with the geo‐positioning of the recorded rangefinder data. To account for this, calibration data was collected regularly and used to spatially adjust the locations using the spatial adjustment in ArcGIS Pro ver. 3.2.0.

#### 2.2.2 Satellite Tracking

Satellite‐tracking data were derived from 11 GPS‐tagged cranes. The tagging of juvenile cranes was undertaken at breeding sites in southcentral (59.73/15.48) and southwestern (57.49/13.35) Sweden during July 2013. Pre‐fledged juveniles were hand‐captured by short distance runs from a car or a hide, and transmitters were attached using flexible harnesses (Månsson et al. 2013). The tagging procedure was approved by the Animal Ethics Committee of central Sweden (C104/10, C53/13). As the juvenile cranes follow the parents during the first autumn migration, the GPS tracks reflect the overall migration paths of the family group. The common cranes were tagged with ARGOS GPS/GSM transmitters (weight 80 g) powered by solar panels. Transmitters recorded GPS positions at 15–30 min intervals during daylight hours. The transmitters had a pre‐set geofence encircling the southern open sea part of the Baltic Sea, within which GPS positions were collected every 30 s. In addition to recording geographic locations as longitude and latitude, the transmitter GPS module also logged altitude, speed over ground and movement direction.

As the study area was entirely located within the geofence, the GPS data (*n* = 1762 location fixes) all had high position‐recording frequency with the GPS receiver retaining information from a previous location fix about satellite position as well as up‐to‐date almanac and ephemeris data. The GPS tracks are shown in Supporting Information S1. Both during spring and autumn, crane crossings only took place during daylight hours. A total of 8 crane crossings were recorded on 5 days during the autumns of 2013 and 2014; during the spring of 2014 and 2015, three crossings took place on separate days.

### 2.3 Meteorological Data

The weather data were extracted from the regional weather model by StormGeo (www.storm.no) to relate obtained satellite and rangefinder tracks to spatiotemporal explicit weather conditions. The regional model is based on the global weather model run by the European Centre for Medium‐Range Weather Forecasts (ECMWF, https://www.ecmwf.int/). The spatial resolution of the WRF model is 0.1 × 0.1 degree, and the temporal resolution is 1 h. Meteorological data for each observation or GPS location were extracted, using the date/time and latitude/longitude of each observation. Meteorological variables included precipitation (mm), clearness (%), humidity (%), air pressure (hPa), wind speed (m/s) and wind direction (degrees). We calculated the relative wind directions for the cranes, which were categorised into four equally distributed (90°) wind classes (head wind, tail wind, left cross wind and right cross wind). The clearness parameter (index from 0% to 100%) reflects the level of solar radiation.

### 2.4 Data Handling

Satellite tracking data underwent some basic filtering. Several GPS positions with erroneous altitude measurements were filtered manually by removing altitudes < 0 m, and measurements that were clearly unrealistic considering the fine temporal resolution of the dataset, that is, records showing clear spikes of more than 100 m over < 3 min. Distance to land in meters was calculated for each observation by calculating the shortest Euclidean distance to digitised coastlines of the region in ArcGIS Pro ver. 3.2.0.

### 2.5 Vertical Distribution Modelling

The vertical distribution of common cranes during migration across the Arkona Basin was modelled by studying the effect of meteorological conditions on the flight altitudes recorded by GPS tracks and laser rangefinder tracks. As relationships were expected to be non‐linear with data having non‐normally distributed errors, we used the semi‐parametric and data‐driven generalised additive mixed modelling approach (GAMMs; Wood 2006; Zuur et al. 2009). This also enabled us to account for the spatial and temporal autocorrelation (non‐independencies in the residuals) in the data.

With flight altitude as the dependent variable, we included the most influential parameters: distance to land (meters), wind speed (m/s) and clearness as smooth functions (*k* = 5). Relative wind direction was added as a factorial variable, where the wind was classified as either head, tail, left, or right‐sided. To account for the temporal autocorrelation in the data, we included a first‐order autocorrelation structure, corAR1, grouped by the individual tracks, as well as the sampling method (GPS/rangefinder) as random effects. Differences in the density of data points between GPS and rangefinder samples were accounted for by resampling the GPS data, which has a much higher density than rangefinder data. Accordingly, the requirement of equal probability of observation of cranes has been met in order for the mixed model to capture the pattern of dependence within the random effects. The model was fitted with a gamma distribution (with log link).

The predictive accuracy of the models was evaluated by using a split sample approach, fitting the model on 70% of the tracks and evaluating the models on the remaining 30%. The agreement between the observed and predicted altitudes was tested using the Spearman's rank correlation coefficient. The model fit was also assessed by the adjusted R‐square values (variance explained) and an inspection of the residuals. The GAMM models were fitted using the ‘mgcv’ package ver. 1 (Wood 2006) in R (version 2.13.0; R Development Core Team 2004).

## 3 Results

### 3.1 Migration Intensity of Common Crane

**FIGURE 2:**
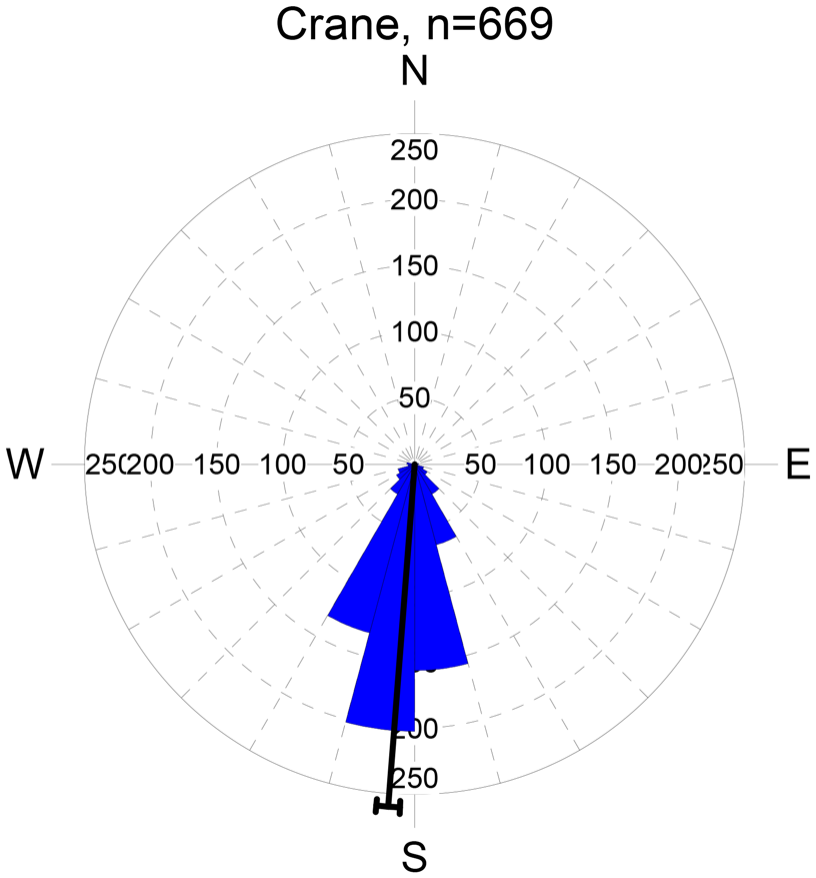
Sampled migration directions of common cranes at Falsterbo, 20–24 September 2013 when a high sample size was obtained at the site. Numbers on the *y*‐axis refer to sample size (number of recordings by laser rangefinder). Sample size (*n*) refers to the number of individuals, albeit they typically occurred in flocks. Each wedge represents a sector of 15°. The mean direction is indicated by the black line running from the centre of the graph to the outer edge. The arcs extending to either side represent the 95% confidence limits of the mean direction.

During both autumn and spring migration, the cranes displayed a strong directional trend (see Figure 2 for an example recorded during autumn migration at Falsterbo). The vast majority of directions recorded by rangefinder from Falsterbo during autumn were concentrated around south (185°) in the direction of Rügen. During spring, the mean direction of migrating cranes was 13°.

### 3.2 Flight Altitude Distribution of Common Crane

**FIGURE 3:**
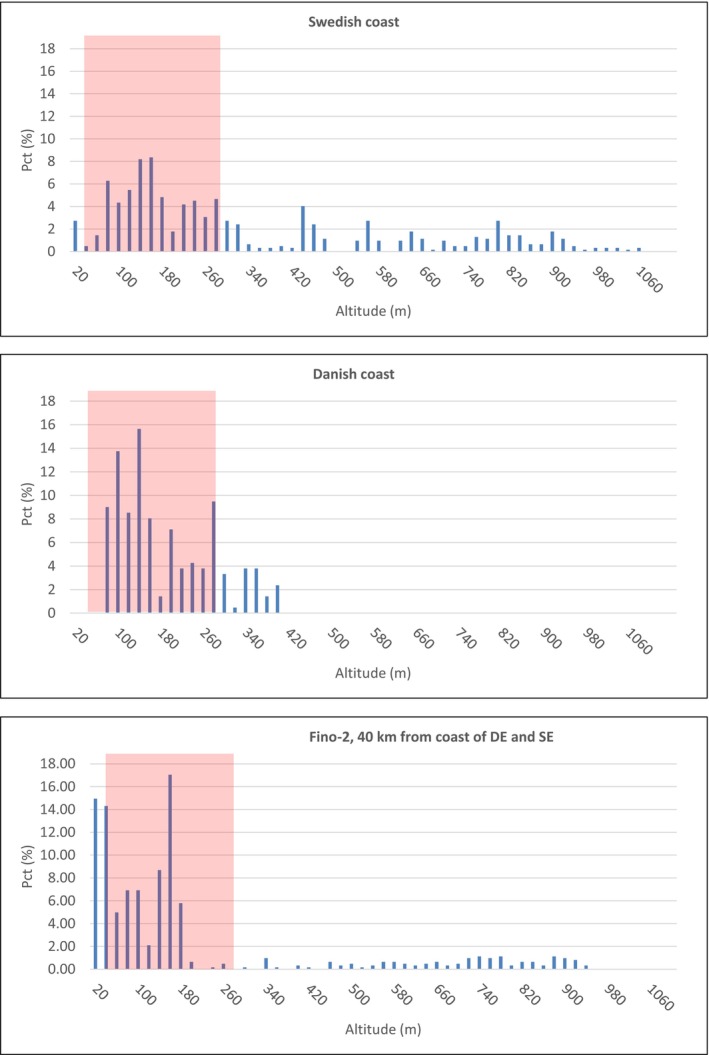
Frequency distribution of altitude measurements of common crane (number of track nodes) by laser rangefinder at the Swedish and the Danish coast and at the FINO‐2 platform during autumn, see Table 1. The track nodes have been aggregated into 20 m bins. The altitude range of the rotor zone of future offshore wind turbines (15 MW) is indicated by red shading (see Supporting information S1 for location of future offshore wind farms in the study area).

**FIGURE 4:**
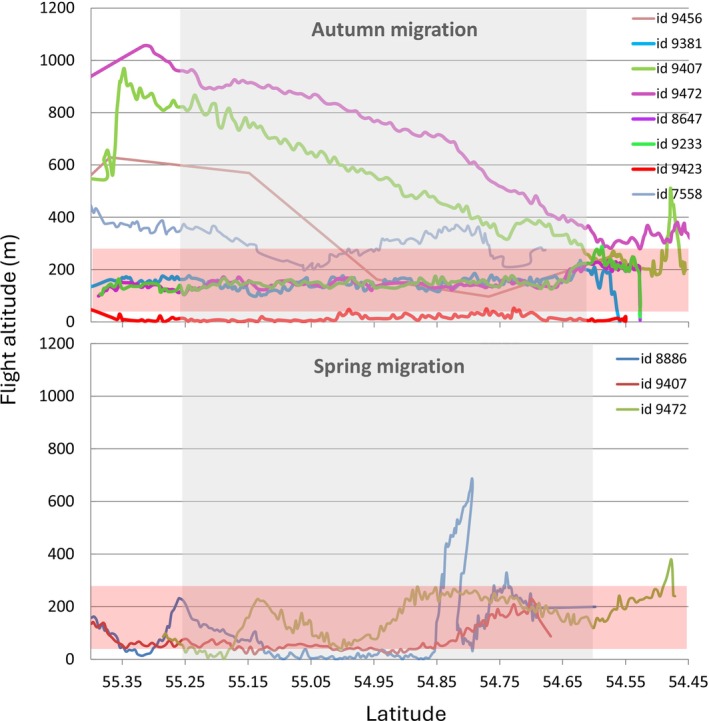
Altitude measurements of 11 GPS‐tagged common crane 2013–2015 plotted against latitude. The location of the Arkona Basin is indicated by the grey‐shaded area. The different colours of the altitude measurements represent different individuals as indicated by the track IDs. The altitude range of the rotor zone of future offshore wind turbines (15 MW) is indicated by red shading (see Supporting information S1 for location of future offshore wind farms in the study area).

The patterns of flight altitude of migrating cranes recorded by rangefinder show that the birds cross the Arkona Basin at variable altitudes from a few metres to more than 200 m above sea surface (Figure 3). The median altitude measurements at the Swedish and Danish coasts were 251.4 and 129.1, respectively. The lower quartile of the flight altitudes at the Swedish coast during the autumn season was 157.7 and 92.7 m at the Danish coast, while the upper quartile of the measured altitudes during the same season was 497.6 and 239.7 m at the Swedish and Danish coasts, respectively. The general descent in flight altitude from the Swedish coast in autumn was nonetheless very clear. The satellite tracking data confirmed the patterns recorded by direct observation (Figure 4). During autumn, four of the eight tracked cranes were recorded over the Swedish coast at flight altitudes above 400 m. The crossing of approximately 80 km of open water took about 1–2 h, depending on weather conditions. As only a few of the tagged birds returned to Sweden for their second and third summers and some of the GPS tags stopped working after the first autumn season, only three satellite tracks were obtained during spring (Figure 4). One individual was able to increase altitude to more than 600 m over a well‐defined shorter distance off the German coast and then continued towards Sweden below 200 m. The two other cranes displayed more distinct descents from the German coast to the offshore parts of the region. One of the birds descended to altitudes between 50 and 250 m, while the other bird descended to below 50 m before reaching the Swedish coast and ascended to 250 m (Figure 4). During autumn, four of the satellite tracked birds displayed clear descents in altitude from the Swedish coast, yet the angle of descent varied greatly between tracks and days. Two tracks crossed the entire open sea area at 175 m altitude and displayed a slight increase in altitude off the German coast (Figure 4). One tracked individual flew just a few meters above water level all the way when crossing the Baltic Sea.

**FIGURE 5:**
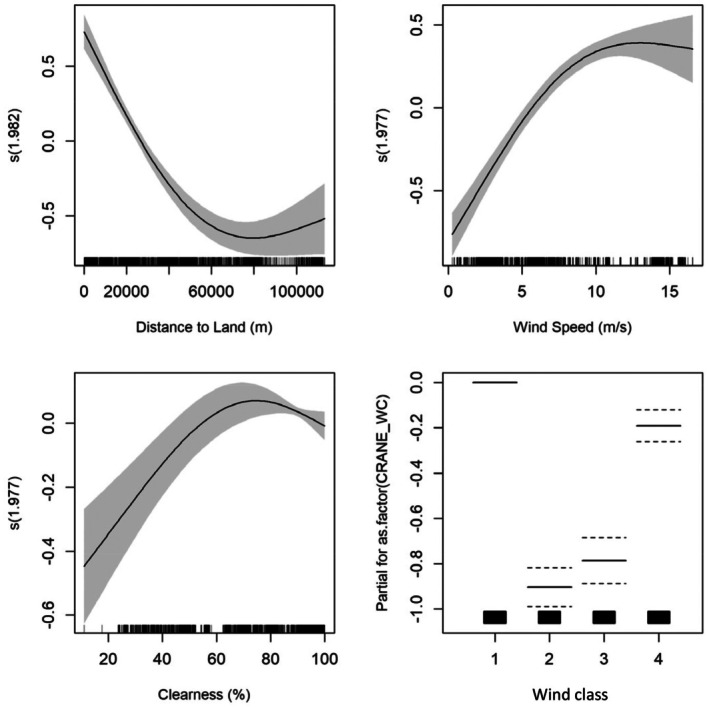
GAMM response curves for the common crane flight altitudes based on satellite tracking and rangefinder data from both spring and autumn collected in the Arkona Basin 2013–2015. The values of the environmental predictors are shown on the *x*‐axis. The response (flight altitude) on the *y*‐axis is on the scale of the linear predictor, which means that the *y*‐axis reflects the contribution of each predictor to the overall linear predictor, rather than the actual predicted values of the response variable. The degree of smoothing is indicated in the title of the *y*‐axis. The shaded areas and the dotted lines show the 95% Bayesian confidence intervals. The four wind classes in the bottom right graphic are (1) headwind, (2) easterly crosswind, (3) westerly crosswind and (4) tailwind.

The predictive accuracy of the GAMM was high, with moderate agreement between observed and predicted altitudes and a Spearman's rank correlation of 0.40 and an adjusted *R* ^2^ of 0.35. The model fit can be regarded as good as we were able to account for the strong temporal and spatial autocorrelation in the track data by using the correlation structure and random term. Based on the confidence intervals, the model was best at predicting intermediate flight altitudes, whereas predictions at very high or low altitudes were less precise (Figure 5).

**FIGURE 6:**
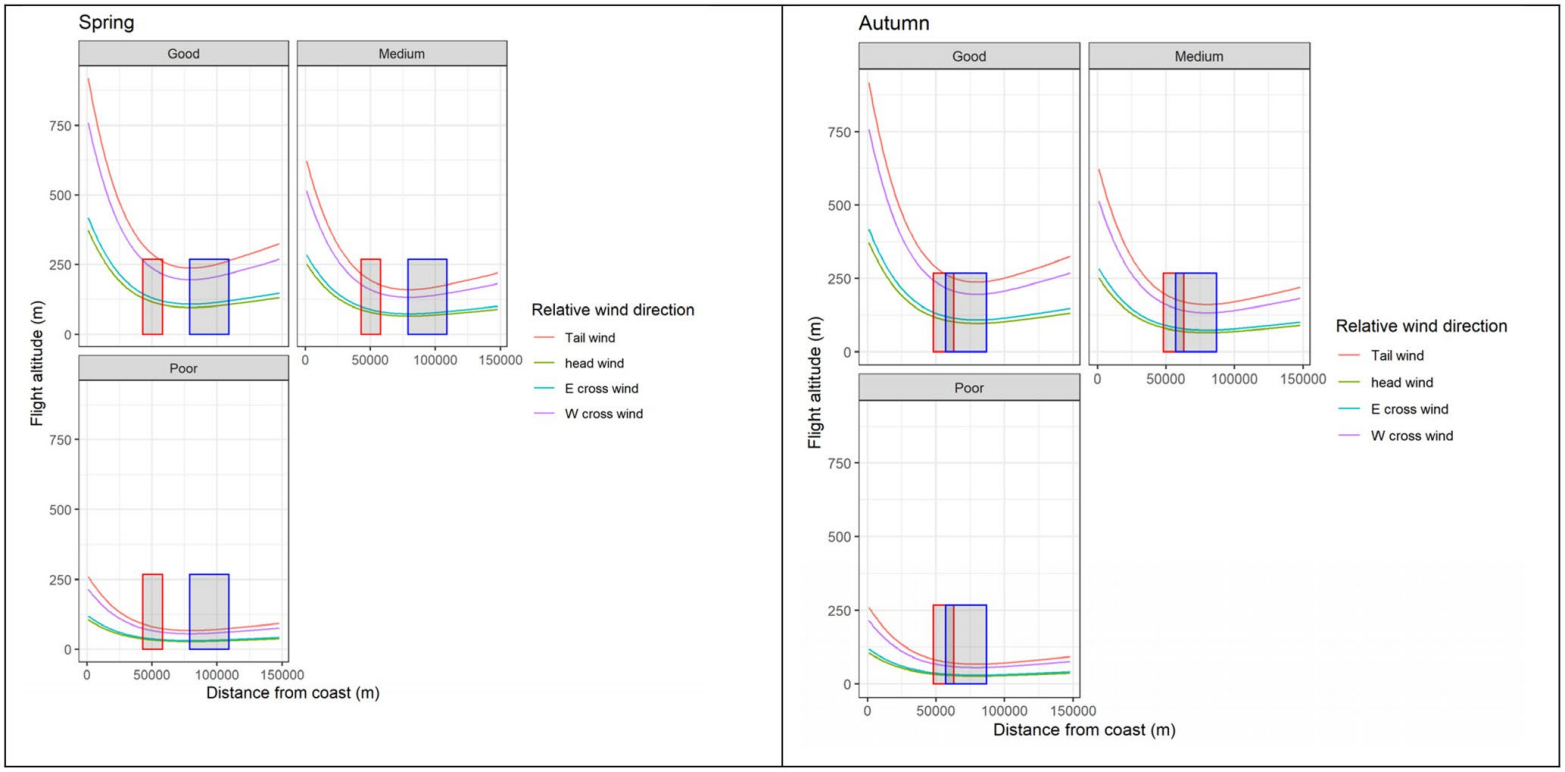
Average predicted altitude for common crane in relation to distance from the coast of Sweden during autumn and from the coast of Germany during spring in different clearness (good, medium, poor) and wind directions as compared to the height of fictitious wind turbines. All other predictor variables are set to mean values. The coloured lines indicate the predicted flight altitudes, and the red and blue rectangles indicate the height of two fictitious wind farms with 15 MW wind turbines placed in the central part of the region.

We used the flight model for predicting the average seasonal flight altitude during average, poor and good clearness and during tail, head and cross winds. According to the predictions, the common cranes descend in altitude after leaving the coast of Germany and Sweden and fly higher in tail winds and clearer weather (Figure 6). During spring, except for during optimal conditions with tail wind and clear weather, the model shows that most common cranes fly below 250 m when they cross the Arkona Basin (Figure 6). Descents from the German coast were predicted in all assessed weather scenarios, with the steepest descent (> 12.0 m/km) in optimal conditions and the flattest descent (> 1.7 m/km) in headwinds and poor clearness (< 60%). During autumn, although optimal conditions may enable birds to soar to altitudes above 700 m before leaving the coast of Sweden, they generally descend to altitudes below 250 m over the central parts of the Arkona Basin. As for the situation during spring migration, the cranes during autumn migration were predicted to fly at rotor height during moderate and poor clearness and during crosswind and headwind conditions. As during spring, cranes were predicted to descend from the coast (Sweden) in all assessed weather scenarios, with the steepest descent (> 12.0 m/km) in optimal conditions and the flattest descent (> 1.6 m/km) in headwinds and poor clearness.

## 4 Discussion

As the first study of the flight altitude of common cranes during seasonal migrations across the Baltic Sea, we have shown how the flight altitude of cranes changes as a function of wind conditions. Although the occurrence of thermals that can provide energy‐free lifts is relatively low at sea (Newton 2008), soaring species like cranes may find favourable wind conditions when crossing large expanses of open sea (Duriez et al. 2018; Nourani et al. 2018, 2021; Škrábal et al. 2023; Pekarsky et al. 2024). Yet, detailed studies of the migratory behaviour of cranes have been missing from sea areas targeted for offshore wind development. Soaring species like the crane migrate in spatially restricted corridors due to a strong avoidance of crossing large expanses of open water (Kerlinger 1989; Leshem and Yom‐Tov 1996). In combination with this spatial constraint, favourable weather conditions further concentrate migration temporally, often resulting in large flocks moving within narrow corridors on specific days (Alerstam 1975). The avoidance of open sea areas during long‐distance migration may be seen as migration strategies to both mitigate the risk of wind drift over open sea during cross wind conditions (Alerstam and Bauer 1973; Alerstam 1975) and the risk of high energetic costs due to the lack of thermals offshore (Newton 2008). These strategies often result in aggregations of migrating cranes and other species of soaring migrants, and increased sensitivity of the migration corridors used by the cranes to cross open sea areas (Gauld et al. 2022).

Our results from both the satellite tracking and the rangefinder data corroborate earlier findings that the cranes use the thermal uplifts occurring near the coast to gain altitude before crossing the Arkona Basin (Mortensen et al. 2020). However, the results provided evidence that the flight altitude of cranes crossing the basin strongly depends on the wind conditions and clearness. Both during the spring and autumn migration, the cranes utilise thermals at the coast to soar to altitudes which frequently reach > 300 m. Despite variability in wind and clearness conditions, the model predictions showed that the mean flight altitude profiles over the basin descended to altitudes below 250 m corresponding to the rotor height of future wind turbines towards the central offshore parts. A steeper descending trend was observed during headwind and during poor clearness. This characteristic may in part be explained by the low wind support during headwind and the known tendency of migrating birds to fly closer to the surface to reduce the effect of overall negative wind support (Alerstam 1990), as wind speeds tend to be lower at lower altitudes.

Despite the general flight altitude patterns, isolated instances of cranes ascending to altitudes above 250 m were observed near the German and Swedish coasts. These events were recorded by two GPS‐tagged individuals during both spring and autumn migration. These two events suggest that cranes may use localised thermals and/or wind support when crossing the Baltic Sea. Similar behaviour has been observed in common cranes passing the Black Sea (Pekarsky et al. 2024), in ospreys ( *Pandion haliaetus* ) crossing the Mediterranean Sea (Duriez et al. 2018), and in red kites crossing the Adriatic Sea (Škrábal et al. 2023). Further studies are recommended to clarify the extent to which cranes utilise localised thermals and horizontal wind support, or both, during sea crossings in the Baltic Sea.

Our study resulted in a large sample and spatial coverage of rangefinder and GPS tracks of migrating cranes crossing the Baltic Sea, and accordingly, the predicted flight height dynamics are judged as being transferable to other parts of the Baltic Sea. Yet it is worth noting the following limitations of the study. As the rangefinder tracking was undertaken by human observers by visual detection, a detection bias cannot be ruled out during tracking of cranes, especially when cranes are flying above 600 m. However, this bias is unlikely to have played an important role as the focus of the study was on the altitudinal range from sea surface to 600 m, which is relevant in relation to offshore wind farms. Detection bias is inevitable during situations with low visibility, yet recordings were not undertaken during such conditions. In total, only 11 GPS tracks of migrating cranes were collected, and even if they provided unique data on the flight altitude of the birds across the entire width of the Baltic Sea, the sample size did not allow for computation of a separate flight altitude model based alone on the GPS data. As both the rangefinder and GPS data were primarily collected during 2 years (2013–2014), uncertainty exists regarding the inter‐annual variation in the dynamics of the flight altitudes of cranes.

As the flight altitude of cranes changes significantly with weather conditions, the probability of interaction and potential collision with offshore wind energy development will most likely vary in the Arkona Basin. The Arkona Basin is currently the focus of large‐scale offshore wind farm development with a total of 27 commissioned, consented and planned offshore wind projects over the next couple of decades (Supporting information S1). As the future 15 MW+ wind turbines deployed in the Arkona Basin will be at least 250 m high, the overall collision mortality will depend on the frequency of adverse conditions which cause the birds to fly at or below 250 m. Despite the weather‐induced variations in flight altitude, these behavioural investigations clearly indicate that the vast majority of cranes will cross the Arkona Basin at altitudes between 50 and 250 m. Accordingly, although cranes may find suitable migration conditions over the open water parts of the Arkona Basin, lower flying altitudes and lower climb rates as compared to the coastal areas are to be expected. Obviously, it is a conservation priority to implement efficient measures to mitigate the risk of collision with the future large‐scale wind energy installations in the region (de Lucas et al. 2012). Requirements for mitigation technologies to enable automated shutdown as a means of protecting birds from colliding with wind turbines are now appearing in many of the world's wind farm development regions. Collision mitigation can be applied as automated or controlled shutdown and can either apply to the entire wind farm or single turbines. Tests of the efficiency of advanced monitoring and shutdown systems have indicated that an efficiency above 80% in terms of reduced mortality rates of large species of birds like eagles may be achieved (McClure et al. 2021).

## Author Contributions

**Henrik Skov:** conceptualization (lead), formal analysis (supporting), methodology (equal), project administration (lead), resources (lead), supervision (lead), validation (supporting), writing – original draft (lead). **Stefan Heinänen:** formal analysis (lead), investigation (equal), methodology (equal), software (equal), writing – review and editing (equal). **Lars O. Mortensen:** formal analysis (equal), software (supporting), writing – review and editing (equal). **Johan Månsson:** investigation (equal), methodology (equal), supervision (equal), writing – review and editing (equal). **Lovisa Nilsson:** investigation (equal), methodology (supporting), supervision (supporting), writing – review and editing (equal). **Rune S. Tjørnløv:** formal analysis (equal), software (supporting), writing – review and editing (equal). **Ramunas Zydelis:** conceptualization (supporting), investigation (lead), methodology (supporting), writing – review and editing (equal).

## Conflicts of Interest

The authors declare no conflicts of interest.

## Supporting information


**Appendix S1:** ece372714‐sup‐0001‐AppendixS1.docx.

## Data Availability

The data that support the findings of this study are openly available in Zenodo at https://doi.org/10.5281/zenodo.17490907.
