# Supplementary material for: Flight Altitude of Common Cranes (Grus grus) Crossing the Arkona Basin (Baltic Sea): Implications for Offshore Wind Farm Development
Source: Ecol Evol. 2025 Dec 12;15(12):e72714. doi: 10.1002/ece3.72714 (PMC12700719; doi:10.1002/ece3.72714)
Supplement: Supplementary file 1 — Appendix S1: ece372714‐sup‐0001‐AppendixS1.docx. [file ECE3-15-e72714-s001.docx]

Skov et al. *Flight altitude of common cranes (Grus grus) crossing the Arkona Basin (Baltic Sea): implications for offshore wind farm development*

**Supplementary information**

**
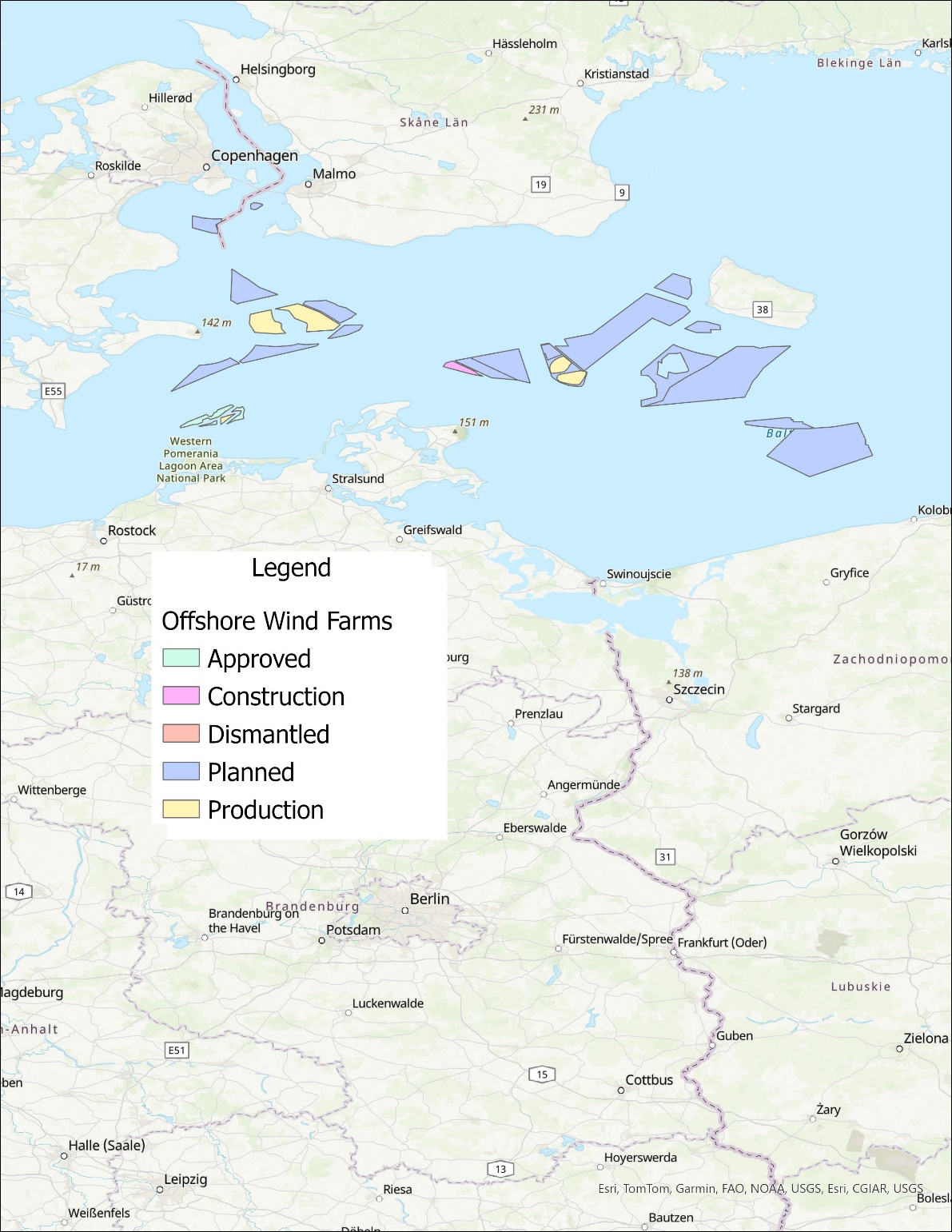
**

Figure S.1 Overview of existing, consented and planned offshore wind farms in the western Baltic Sea.

Figure S.2 GPS tracks of eleven common cranes crossing the Arkona Basin during autumn 2013-2014 and spring 2014-2015. Each dot represents a positional fix. Please note that two birds flew together during autumn 2013.
